# Sources of human infection by *Salmonella enterica* serotype Javiana: A systematic review

**DOI:** 10.1371/journal.pone.0222108

**Published:** 2019-09-03

**Authors:** Nabanita Mukherjee, Vikki G. Nolan, John R. Dunn, Pratik Banerjee

**Affiliations:** 1 Division of Epidemiology, Biostatistics, and Environmental Health, School of Public Health, University of Memphis, Memphis, Tennessee, United States of America; 2 Communicable and Environmental Diseases and Emergency Preparedness, Tennessee Department of Health, Nashville, Tennessee, United States of America; University of Connecticut, UNITED STATES

## Abstract

Non-typhoidal *Salmonella* (NTS) infection is one of the major causes of diarrheal disease throughout the world. In recent years, an increase in human *S*. Javiana infection has been reported from the southern part of the United States. However, the sources and routes of transmission of this *Salmonella* serotype are not well understood. The objective of this study was to perform a systematic review of the literature to identify risk factors for human *S*. Javiana infection. Using PRISMA guidelines, we conducted a systematic search in Web of Science, PubMed, and the Morbidity and Mortality Weekly Report (MMWR). Searches returned 63 potential articles, of which 12 articles met all eligibility criteria and were included in this review. A review of the literature indicated that both food and non-food (such as animal contact) exposures are responsible for the transmission of *S*. Javiana infection to humans. Consumption of fresh produce (tomatoes and watermelons), herbs (paprika-spice), dairy products (cheese), drinking contaminated well water and animal contact were associated with human *S*. Javiana infections. Based on the findings of this study, control of human *S*. Javiana infection should include three factors, (a) consumption of drinking water after treatment, (b) safe animal contact, and (c) safe food processing and handling procedures. The risk factors of *S*. Javiana infections identified in the current study provide helpful insight into the major vehicles of transmission of *S*. Javiana. Eventually, this will help to improve the risk management of this *Salmonella* serotype to reduce the overall burden of NTS infection in humans.

## Introduction

Nontyphoidal *Salmonella* (NTS) infection or salmonellosis is a major public health concern as millions of human cases are reported every year throughout the world. Approximately 93.8 million cases of gastroenteritis are estimated to occur each year globally due to NTS infections, a majority of which are foodborne (estimated at 80.3 million cases per year) [1]. In the United States, NTS infections are reported to cause 1.2 million cases of foodborne illness annually [2]. Even though NTS infections cause only 11% of foodborne diseases, it is responsible for approximately 35% of hospitalizations and 28% of deaths [2]. NTS infection in humans is caused by *Salmonella enterica* species [3]. *S*. *enterica* subsp. *enterica* serovar Javiana (commonly known as *S*. Javiana) belongs to D1 (O:9) serogroup and is a highly virulent serotype [4]. According to USDA’s Food Safety and Inspection Service (FSIS) and FoodNet data, *S*. Javiana is the fourth most common *Salmonella* serotype that is associated with NTS in the USA responsible for 5% of overall salmonellosis cases [5–7]. *S*. Javiana produces typhoid toxin or *Salmonella* cytolethal distending toxin (CDT) [8]. This trait distinguishes *S*. Javiana from most other *Salmonella* serotypes. The genotoxin CDT is one of the major virulence factors of *S*. Typhi, which causes typhoid fever in humans, but not commonly found in major NTS-causing serotypes including *S*. Enteritidis, *S*. Typhimurium, *S*. Newport, and *S*. Heidelberg which are among the top five serotypes causing this disease [9]. *S*. Javiana strains carry the genetic assembly including *cdtB*, *pltA*, and *pltB* that encode the CDT and this toxin plays an important role in DNA damage and systemic host colonization by this serotype [9, 10].

The symptoms of *S*. Javiana infection include diarrhea, fever, and abdominal cramps [11, 12]. Usually, illnesses caused by *S*. Javiana are self-limited. However, in some cases, severe health conditions, including liver abscess, meningitis, and cholecystitis with gallbladder perforation have been reported [13–15]. Although rare, it can cause bloodstream infections in immunocompromised individuals [16]. In general, the most vulnerable population to NTS infection includes the children aged below four years, elderly adults, pregnant women, and immunocompromised individuals. Likewise, *S*. Javiana infection predominantly occurs in infants and children of ages less than four years [17]. The case fatality rates of *S*. Javiana infection is 38% in children and 47% in adults [18, 19].

*S*. Javiana infection has been reported worldwide, including USA, Germany, and Australia [20–22]. Since 1996, the overall incidence of *S*. Javiana related illnesses has increased substantially in the United States [23]. For example, in 2001, a seven-fold increase of *S*. Javiana cases (n = 43) was reported in Mississippi compared to the previous year [24]. More recently, episodes of *S*. Javiana cases were reported from several US states including, Arkansas, Connecticut, Florida, Georgia, Illinois, Maryland, Michigan, Minnesota, Mississippi, Missouri, New Mexico, North Carolina, Oregon, South Carolina, Tennessee, and Wisconsin [17, 22]. The geographic distribution of the *S*. Javiana serotype suggests similar sources of the infection, such as local foods, distribution of local food products, and/or natural reservoirs. Although *S*. Javiana cases can occur during any season, it has been mostly observed during July through October [17]. This seasonal trend of *S*. Javiana related illnesses may be related to the seasonality of amphibian as well as reptile life cycles [25]. Certain amphibians such as frogs and toads increase their populations during this time. Their abundance in the environment may facilitate the spreading of *S*. Javiana. Although several risk factors and sources may promote the spread of *S*. Javiana, the most common risk factors are still not well established. This systematic review made an extensive effort to identify the most common sources of *S*. Javiana infection in human based on scientific literature search as of July 1, 2018.

## Methods

This review was conducted following the guidelines outlined in the Preferred Reporting Items for Systematic Reviews and Meta-Analyses: The (PRISMA) Statement [26] (S1 Fig).

### Search strategy

A systematic search was conducted using the electronic databases Web of Science, PubMed, and Morbidity and Mortality Weekly Report (MMWR) to identify the relevant articles included in this review [27]. The primary search included the keyword “*Salmonella* Javiana” in the Web of Science database. A repeat search was conducted using the same keyword in PubMed, as well as MMWR databases, to ensure capturing all relevant articles to be included in this review. Additionally, the articles that were referenced in the bibliography of the identified articles were thoroughly reviewed for relevance. Following this, EndNote bibliographic software was used to remove duplicate references.

### Eligibility criteria

Articles that were considered eligible for this review met the following criteria: 1) written in English, 2) reports of human NTS infection caused by *Salmonella* Javiana, 3) original articles published as of July 1, 2018, and 4) case reports were included only when the authors identified a source of the *S*. Javiana infection.

### Exclusion criteria

This systematic review did not include abstracts, summary, and review articles. Documents that have not been published in a peer-reviewed scientific journal were not included in this systematic review.

### Study selection

The titles, abstracts, and keywords of the articles were screened thoroughly to ensure the consistency with the inclusion and exclusion eligibility criteria. All full-text articles were screened to identify the sources of *S*. Javiana infection in human. Only articles that studied laboratory-confirmed NTS infection caused by *S*. Javiana serotype were included in this review. Articles in which NTS infection was suspected but not confirmed were excluded from the review.

### Data collection process and data items

The number of reported cases, gender, and age of the subjects, exposure information, as well as the presence or absence of the comparison group, are presented in this systematic review.

### Quality assessment and risk of bias analysis

For the studies included in this systematic review, a thorough assessment of the methodological quality and risk of bias analysis was performed by two authors (NM and PB) independently. The Newcastle-Ottawa Scale (NOS) was used for case-control studies [28, 29], while the case series studies were appraised using National Heart, Lung, and Blood Institute (NHLBI) Study Quality Assessment Tools [30, 31]. NOS for case-control studies included three criteria 1) Selection (which was evaluated based on a) if the case definition was adequate, b) representativeness of the cases, c) selection of controls and d) definition of controls; 2) Comparability (evaluated based on the comparability of case and controls in terms of study design and data analysis); 3) Exposure (assessed by a) ascertainment of exposure, b) whether or not the same method of ascertainment was applied for cases and controls, and c) non-response rate). The NHLBI Tool for case series studies included 1) Was the study question or objective clearly stated; 2) Was the study population clearly and fully described, including a case definition; 3) Were the cases consecutive; 4) Were the subjects comparable; 5) Was the intervention clearly described; 6) Were the outcome measures clearly defined, valid, reliable, and implemented consistently across all study participants; 7) Was the length of follow-up adequate; 8) Were the statistical methods well-described; 9) Were the results well-described. The best evidence synthesis was performed according to NIH and NHLBI guidelines by rating study qualities into three bins: good, fair, and poor [32].

## Results

### Search results

**Fig 1:**
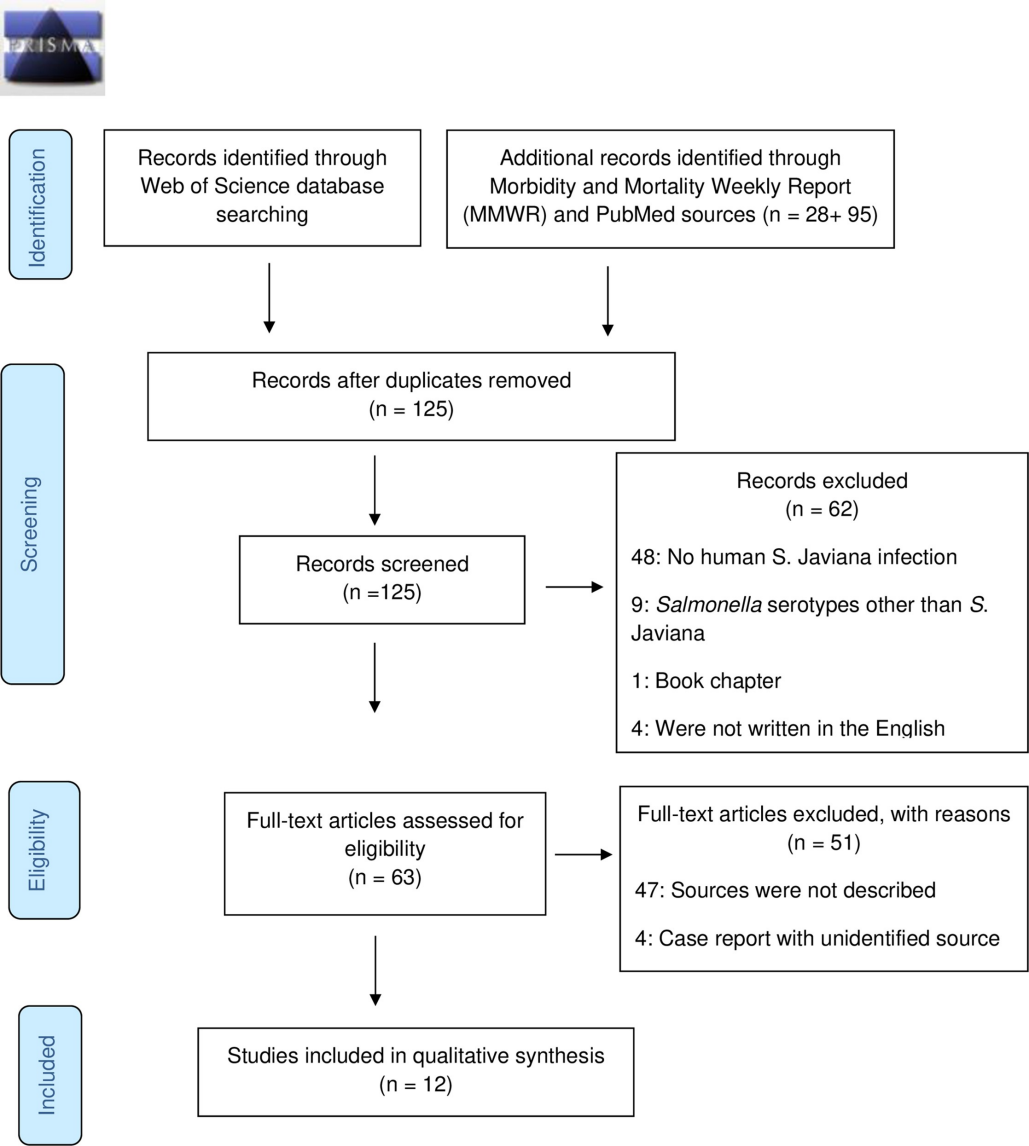
PRISMA flow diagram. Flowchart of articles included in this systematic review.

The initial search yielded a total of 219 articles from Web of Science, PubMed, and MMWR databases. A total of 94 duplicates were removed using EndNote bibliographic software. Upon removal of duplicates, a thorough primary screening of titles, abstracts, and keywords were performed yielding a total number of 125 articles. Of these 125 articles, 62 were excluded, because, a) 48 articles pointed out the presence of *S*. Javiana infection in plants, seafood, and animals, b) nine reported NTS infection caused by *Salmonella* serotypes excluding *S*. Javiana, c) one was a book chapter and d) four articles were not written in English; 63 articles remained for review. Upon reviewing the full text of the 63 articles, 51 were excluded. Forty-seven did not describe the source of the *S*. Javiana infection, and four were case reports with unidentified sources of infection. Finally, 12 articles met all eligibility criteria and were included for this systematic review (Fig 1).

### Study characteristics

Eleven of 12 studies reported on *S*. Javiana infections occurring in the United States [6, 11, 22, 24, 33–39] and one reported on cases in Germany [21] (Table 1). One of the 11 USA studies included cases from Canada as well [35]. It is noteworthy that four out of 11 USA studies reported multistate outbreaks of *S*. Javiana infection [6, 35, 37, 38]. Of the 12 studies, three identified tomatoes [35, 37, 39] and two identified cheese [11, 33] as the potential vehicles. Paprika-spiced potato chips [21] and watermelon [34] were also associated with *S*. Javiana infection in humans. Non-foodborne sources including drinking well water [6], exposure to reptiles or amphibians [20], and animal feeding operations [28] were significantly associated with the *S*. Javiana infection. Finally, S. Javiana infection was also found to be a healthcare-associated infection in two instances [22, 36]. *S*. Javiana was found to be the most predominant serotype in a tertiary care center[22]. In addition, *S*. Javiana associated outbreak [36] was reported from hospital setting, and the potential source of the outbreak was considered to be the contaminated food by food handlers.

**Table 1:** Summary of risk factors associated with *S*. Javiana infections.

Authors	Study title	Design	Quality (score)	Study period	Subjects	Study location	Exposure
Hedberg et al.[11]	A Multistate Outbreak of *Salmonella* Javiana and *Salmonella* Oranienburg Infections due to Consumption of Contaminated Cheese	Case-control	Good^1^ (8)	May and June 1989	In case-control study I, cases = 31, and matched controls = 60. In case-control study II, cases = 50, community controls = 100, and healthy family members controls = 64.	MN, USA	Consumption of Cheese
Alley and Pijoan[33]	*Salmonella* Javiana Food Infection	Case series	Good^2^ (8)	July 1942	N = 40	NM, USA	Consumption of Cheese
Lehmacher et al.[21]	Nationwide Outbreak of Human Salmonellosis in Germany due to Contaminated Paprika and Paprika-powdered Potato Chips	Case series	Good^2^ (7)	April to July 1993	N = 1000 (estimated cases)	Germany	Consumption of paprika or paprika-spiced potato chips
Blostein[34]	An Outbreak of *Salmonella* Javiana Associated with Consumption of Watermelon	Case-control	Good^1^ (7)	June 1991	N = 57	MI, USA	Consumption of watermelon
Corby et al.[35]	Outbreaks of *Salmonella* Infections Associated with Eating Roma Tomatoes—United States and Canada, 2004	Case-control	Good^1^ (7)	July 2004	In USA study, N = 106; cases = 53, and controls = 53; In Canada study, N = 7	MD, MI, MO, NC, NH, OH, PA, VA, WV of USA and ON, CA	Ingestion of Roma tomatoes
Hedberg et al.[37]	Outbreaks of Salmonellosis Associated with Eating Uncooked Tomatoes: Implications for Public Health	Case-control	Fair^1^ (6)	June through August 1990	N = 176; In MN study, case = 32, and control = 34. In MI study, case = 12, and control = 12.	MN, MI, IL, and WI of USA	Consumption of tomatoes
Srikantiah et al.[39]	Web-based Investigation of Multistate Salmonellosis Outbreak	Case-control	Fair^1^ (6)	June to July, 2002	N = 82 cases responded to the survey	FL, USA	Ingestion of foods containing diced Roma tomatoes
Clarkson et al.[6]	Sporadic *Salmonella* enterica serotype Javiana Infections in Georgia and Tennessee: A Hypothesis-generating Study	Case-control	Fair^1^ (6)	August to October 2004	N = 896; 72 cases and 824 controls	GA and TN, USA	Drinking well water, and contact with reptiles or amphibians	
Srikantiah et al.[24]	*Salmonella enterica* serotype Javiana Infections Associated with Amphibian Contact, Mississippi, 2001	Case-control	Good^1^ (8)	August to September 2001	N = 164; 55 cases, and 109 controls	MS, USA	Contact with pet and other animal	
Shaw et al.[38]	Presence of Animal Feeding Operations and Community Socioeconomic Factors Impact Salmonellosis Incidence Rates: An Ecological Analysis Using Data from the Foodborne Diseases Active Surveillance Network (FoodNet), 2004–2010	Case series	Good^2^ (7)	2004 to 2010	N = 14,297	CT, GA, MD, MN, NM, OR, and TN, USA	Presence of broiler chicken operations
Rathore et al.[22]	Epidemiology of Nontyphoidal Salmonellae at a Tertiary Care Center in Northeast Florida	Case series	Fair^2^ (6)	1986 to 1992	N = 433 human NTS isolates. *S*. Javiana = 126.	FL, USA	Hospital environment exposure
Elward et al.[36]	Outbreak of *Salmonella* Javiana Infection at a Children’s Hospital	Case-control	Good^1^ (8)	May through June, 2003	N = 205; Cases = 101 and controls = 104.	MO, USA.	Consumption of salad bar foods at the cafeteria of the hospital

Note: Study quality appraisal tools:

^1.^NOS

^2.^NIHLB.

### Study quality and risk of bias assessment

Eight of 12 studies were rated as “good” with quality scores >7. Four studies were rated as “fair” (quality score = 6). The details of the study design and the quality are presented in Table 1. The studies that were rated as “good” were studies with the least risk of bias, and results can be deemed as valid. Studies with some risk of potential bias but with the non-significant risk of invalidity were rated as fair.

### Individual study results

#### Role of cheese in *S*. Javiana infection

There were two studies that revealed an association of human *S*. Javiana infection with the consumption of cheese [11], [33]. Hedberg et al. reported a *Salmonella* outbreak, predominantly caused by laboratory-confirmed *S*. Javiana serotype (n = 136), during May through October of 1989 in Minnesota, USA [11]. To identify the source of the outbreak, Hedberg et al. conducted two separate case-control studies [11]. The first case-control study included those cases who had disease onset from May 1 through May 25, 1989. A total of 31 cases and 60 matched controls were selected in the case-control study I. Both cases and matched controls were interviewed over the telephone to inquire about the food items and beverages they consumed between 12 to 48 hours before the disease onset of the cases. Cases were more likely to have consumed cheese compared to the controls [OR = 4.8 (95% CI 0.9, 33.1) *P* = 0.03]. In addition, specifically, brand A mozzarella cheese [OR = 13.7 (95% CI 1.3, 14.8) *P* = 0.03], mozzarella cheese other than brand A [OR = 7.1 (95% CI 1.1, 46.8) *P* = 0.04], any cheese from salad bar [OR = 21.4 (95% CI 2.1, 217.6) *P* = 0.01] and any processed American cheese [OR = 4.5 (95% CI 1.5, 11.5) *P* = 0.005] were independently associated with infection. The second case-control study included 50 cases, who became ill after May 25, 1989 100 community controls and 64 healthy family members. Both cases and controls were interviewed over the telephone asked about the food items and beverages that they consumed between 48 to 72 hours prior to the disease onset of the cases. The participants were asked to provide the type, brand and barcode numbers and the package label of the cheese that was consumed in the house. In the event that the study subjects reported eating cheese in restaurants, the restaurants were contacted to collect information regarding the types and sources of cheese used in those food items. Cheese consumption, including brand A processed American cheese spread [OR = 8.0 (95% CI 0.9, 71.0) *P* = 0.06], and cheese from salad bar [OR = 4.7 (95% CI 1.2, 18.0) *P* = 0.02], were independently associated with infection. The study found that 32% of the cases (n = 16) but only 8% of the controls (n = 8) consumed cheese from plant X [OR = 7.2 (95% CI 1.7, 33.2) *P* = 0.002]. Likewise, the cases were more likely to have cheese from the shredding facility that received the cheese from plant X [OR = 5.3 (95% CI 1.4, 22.2) *P* = 0.005]. Consumption of cheese from the salad bar was the only variable found to be independently associated with the infection [OR = 12.0 (95% CI 1.4, 99.5) *P* = 0.02]. The presence of *S*. Javiana was found in sealed packages of mozzarella cheese manufactured at plant X during the outbreak period. The second case-control study found that the cheese products responsible for the outbreak were either manufactured in plant X or shredded in any of the four processing plants that received the cheese from plant X. Furthermore, the information from the cheese distributors and manufacturers, collected with the help of Minnesota Department of Health and Minnesota Department of Agriculture, revealed that the outbreak was geographically isolated to the areas where the distribution of cheese happened. The outbreak ended after restricting the supply of cheese from Plant X.

Alley and Pijoan [33] identified cheese as the source of *S*. Javiana outbreak in 1942. The outbreak occurred among a group of Navajo Indians, who consumed cottage cheese from the same local cheesemonger, at Puertocito in New Mexico. A total of 40 patients were suffering from enteritis, some of whom were diagnosed with septicemia. The source of this outbreak was found to be cottage cheese that was produced by a Mexican family. The cheese samples, the fermentation starter, blood samples from the cows whose milk was used to prepare cheese and the blood samples from the family members were collected for laboratory testing. Alley and Pijoan [33] identified that the first enteritis infection occurred in one of the family members of the cheesemonger before the outbreak started. Agglutination testing of the cheesemonger’s family member and the cows revealed that the family member had a high agglutination titer of the *S*. Javiana antigen, but the cows were negative. The cheesemonger used a starter product to prepare cheese, which was prepared from beef pancreas during the winter season of 1941 to 1942. The sample of the starter product, as well as the cheese, made using the starter ferment revealed the presence of the enteritis causative agent. The outbreak came to an end at the time when the consumption of the cheese from the cheesemonger was restricted.

#### Role of spice in *S*. Javiana infection

Lehmacher et al. [21] reported a nationwide outbreak of salmonellosis associated with consumption of paprika and paprika-spiced potato chips in Germany in 1993. There were an estimated 1000 cases, and Children were the primarily affected group with those aged 1–4 years (42.5%) most commonly affected, followed by 5–14 years (26.0%). There were 14 cases (3.3%) aged less than one year [21]. The paprika spice was collected from the producer for lab testing. Laboratory tests identified, eleven serotypes including, *S*. Javiana, *S*. Rubislaw, and *S*. Saintpaul, in the collected paprika powder, paprika spice mixtures, paprika-spiced potato chips, and the patients who consumed paprika-spiced potato chips during that time. The paprika-spiced potato chips were recalled, and the public was advised not to consume those potato chips, effectively ending the outbreak.

#### Role of watermelon in *S*. Javiana infection

Blostein 1993 [34] identified watermelon as the source of *S*. Javiana outbreak in Michigan, USA. *S*. Javiana infection was identified mostly in children from the same elementary school and kindergarten. Most of the infected persons attended in either or both an indoor picnic on June 11, 1991, or a party in the school on June 12, 1991. To identify the source of the outbreak, a study was carried out among the Kindergarten’s students, staff, and visitors who attended one or both events. Fifty-seven persons were interviewed over the telephone to inquire about the illness experience and food exposures during those days. Out of the 57 interviewed persons, 21 were diagnosed with culture-confirmed *S*. Javiana infection, and, thus, were selected as cases in the study. Consumption of watermelon at the event on June 11 was significantly associated with the *S*. Javiana infection [RR = 4.0 (95% CI 1.1, 15.0)]. No other food items exhibited an association with the outcome.

#### Role of tomato in *S*. Javiana infection

Three studies revealed a significant association of *S*. Javiana infection with the consumption of raw tomatoes [35, 37, 39]. In 2005, Corby et al. [35] reported an outbreak of *S*. Javiana infection associated with consumption of Roma tomatoes in nine states of the USA and one province of Canada. A total of 429 laboratory-confirmed salmonellosis cases were identified in a multi-serotype *Salmonella* outbreak in the United States. *Salmonella* Javiana (n = 383, 89.3% of total cases) was the predominantly identified serotype among the patients in this outbreak. The age range of the patients was one through 81 years (median age = 35 years). Forty-eight percent of patients were female. A case-control study was conducted selecting 53 cases and 53 well meal-companion controls by the state and local health departments in collaboration with the Centers for Disease Control (CDC). Forty-seven of 53 (90%) cases consumed Roma tomatoes, and 24 of 53 (48%) controls consumed Roma tomatoes. The multivariable analysis found that the consumption of Roma tomatoes was strongly associated with the *S*. Javiana infection [OR_adj_ = 7.1 (95% CI 1.5, 34.0)].

Hedberg et al. [37] reported on a multistate outbreak of *S*. Javiana infection occurring in 1990 that was also associated with the consumption of raw tomatoes. A total of 176 culture-confirmed *S*. Javiana infections were reported from Minnesota, Illinois, Michigan and Wisconsin from June 28 through August 24, 1990. Most of the cases were female (n = 94, 53%). The age range of the cases was four months to 86 years, with a median age of 35 years. Two independent case-control studies were performed as a part of the epidemiologic investigation of the outbreak by the Minnesota Department of Health and Michigan Department of Public Health. Cases were those individuals diagnosed with culture-confirmed *S*. Javiana infection between June through July 1990. Controls were matched based on the telephone exchange, gender, and age of the cases. In both studies, interviews were performed over the telephone following administration of a standard food questionnaire regarding the consumption of food five days prior to disease onset of cases and the reference time for the controls. Cases, whose family members had a history of diarrhea two weeks before cases’ illness, were eliminated from both studies. The authors did not mention whether they use any such exclusion criteria to select control groups in their study. Moreover, in this paper, the authors did not clarify whether they used any exclusion criteria to select control groups. In the Minnesota case-control study, the age-matched controls were selected based on age ±5 years for the cases who were 19 years or younger and the ±10 years for the cases who were 20 years or older. A total of 32 cases and 34 controls were enrolled in the Minnesota case-control study. Initial bivariable analyses in the Minnesota case-control study showed that tomato consumption was associated with the *S*. Javiana infection with cases being more likely to have consumed tomatoes compared to controls [OR _matched_ = 7.5 (95% CI 1.7, 32.8)]. In the same case-control study, cases were more likely to have tomatoes in restaurants compared to controls [OR _matched_ = 6.3 (95% CI 1.9, 21.4) *P* = 0.001]. The implicated tomatoes, which were consumed by a majority (62%) of the cases, either in the restaurant or from the grocery store was found to have come from a South Carolina based tomato packer. In the Michigan case-control study, matched controls were selected based on ±5 years of case’s age. A total of 12 cases and 12 controls were enrolled. Cases were more likely to have tomatoes in restaurants compared to controls [OR _matched_ = 10.0 (95% CI 1.1, 118) *P* = 0.01]. In both studies, follow up interviews were conducted among cases and controls to collect detailed information about tomato consumption. Consumption of tomatoes in restaurants showed a significant association with the *S*. Javiana infection in both studies. Combining both studies, cases were more likely to have tomatoes in restaurants compared to controls [OR = 8.1 (95% CI 2.9, 23.1) *P* <0.001].

Srikantiah et al. identified pre-diced tomatoes as the source of transmission of a 2002 multistate NTS outbreak [39]. To identify the potential sources of this outbreak, Srikantiah et al. [39] conducted a web-based cohort study of 1,100 attendees of the 2002 USA Transplant Games. A total of 369 (34%) people responded to the survey and among them, 82 (22%) people reported *S*. Javiana infections. Approximately 59% (n = 48) of the ill respondents were transplant recipients. The majorities (53%) of the ill respondents were females, and their age range was four to 71 years with the median age 47 years. To identify the potential source of the outbreak, a follow up web-based case-control study was performed among the 369 survey respondents of the first survey. Of 222 who responded, 217 provided valid responses. Forty-one were diagnosed with diarrhea in between June 25 to July 7 in 2002, and, thus, included as cases and the remaining 176 persons were disease-free and served as controls. Cases were asked about the consumption of foods in the three days before disease onset. The controls were asked the same questions, but regarding the middle three days of the Transplant Games (June 26 through June 28, 2002). Bivariable analyses revealed that the cases were more likely to have consumed foods containing diced Roma tomatoes compared to controls [OR = 4.3 (95% CI 2.1, 9.1) *P* <0.0001]. Diced Roma tomato was the only food item that remained significantly associated with *S*. Javiana infection in the multivariable logistic regression model adjusted for all other food sources.

#### Role of animal contact in *S*. Javiana infection

Clarkson et al. [6] conducted a case-control study to investigate the source of *S*. Javiana infection among humans in sporadic cases in Georgia and Tennessee. Of 117 *S*. Javiana case-patients, 72 (62%) were enrolled in the Clarkson et al. study [6]. Cases were those patients who were diagnosed with culture-confirmed *S*. Javiana infection during August through October 2004, and controls were selected from the respondents of FoodNet Population Survey, conducted from Georgia and Tennessee in 2002 and 2003. The FoodNet Population Survey was conducted over the telephone on a group of volunteers from FoodNet sites. A total of 72 cases and 824 controls were enrolled in the study. The cases and controls were questioned regarding consumption of food, sources of drinking water, and exposure to an animal in a week prior to the disease onset. Cases were more likely to be male compared to controls and younger than 13 years. The median age of cases was five years, and controls were 41 years. Cases were less likely to have consumed tomatoes [OR = 0.3 (95% CI 0.2, 0.5)], poultry products [OR = 0.5 (95% CI 0.2, 0.9)], and shell eggs [OR = 0.6 (95% CI 0.4, 1.1)]. However, cases were more likely to have been exposed to reptiles or amphibians [OR = 2.6 (95% CI 1.2, 5.9)] compared to controls. Cases were more likely to have consumed well water [OR = 3.6 (95% CI 1.9, 6.8)] or to have swallowed water while participating in recreational water activities [OR = 9.5 (95% CI 1.6, 57.9)] compared to controls. Logistic regression models were used to determine the association of several food items and environmental exposures with the *S*. Javiana infection, after controlling for age, gender, and living in rural areas. Multivariable analysis revealed that consumption of tomatoes [OR_adj_ = 0.5 (95% CI 0.3, 0.9) *P* = 0.05] and poultry items [OR_adj_ = 0.5 (95% CI 0.2, 1.0) *P* = 0.06] were protective of *S*. Javiana infection. Drinking well water [OR_adj_ = 4.3 (95% CI 1.6, 11.2) *P* = 0.05], and contact with reptiles or amphibians [OR_adj_ = 2.6 (95% CI 0.9, 7.1) *P* = 0.06] was associated with an increased risk of *S*. Javiana infection.

Srikantiah et al. [24] conducted a matched case-control study consisting of 55 cases and 104 age-matched controls to identify the source of *S*. Javiana infections that occurred during the summer of 2001 in Mississippi, USA. For the cases aged less than five years, controls were randomly selected using the Mississippi State Birth Registry and were matched by age and the county of residence. Cases aged five years and older, random digit dialing was used to select controls and were matched to the cases age group as well as the county of residence. For cases aged six to twenty years, the controls were frequency-matched within two years of case’s age, and for cases 21 years old, controls were frequency-matched within five years of case’s age. Cases were predominantly female, and a majority of the cases were from the greater Jackson area. The age range of the cases was three months to 70 years, with the median age of 24 months. *S*. Javiana infection was evenly distributed over the two months (August through September 2001) period. The matched bivariable analyses revealed that the cases were more likely to have consumed orange juice [OR _matched_ = 2.9 (95% CI 1.2, 6.8) *P* = 0.02] compared to control groups. Cases were also more likely to have had watermelon [OR _matched_ = 9.1 (95% CI 1.1, 78.4) *P* = 0.05] compared to control groups. Other food exposures, such as meat products and eggs, were not associated with the *S*. Javiana infection. Cases were also more likely to have visited a lake or pond [OR _matched_ = 2.8 (95% CI 1.3, 5.8) *P* = 0.006], to have been exposed to snakes [OR _matched_ = 7.3 (95% CI 1.5, 34.7) *P* = 0.01], turtles [OR _matched_ = 6.2 (95% CI 1.7, 22.7) *P* = 0.006], and frogs or toads [OR _matched_ = 2.6 (95% CI 1.4, 4.9) *P* = 0.004] on their property or in the yard compared to control groups. The multivariable analysis also showed exposure to frogs or toads [OR _matched_ = 2.5 (95% CI 1.2, 5.6) *P* = 0.02] was associated with *S*. Javiana infection. Eight of ten cases who were exposed to turtles had also been exposed to frogs or toads. The presence of amphibian in the environment was considered as potential sources of *S*. Javiana infection.

Recently, Shaw et al. [38] investigated the role of agricultural, environmental as well as socioeconomic factors in the occurrence of *Salmonella* infection cases. Data for this study were collected from FoodNet reports from seven states, including Connecticut, Georgia, Maryland, Minnesota, New Mexico, Oregon, and Tennessee. The socioeconomic, environmental and animal feeding operation data were collected from the 2010 Census of Population and Housing (USA Census Bureau, 2010), 2011 American Community Survey (USA Census Bureau, 2011) and the 2007 USA Census of Agriculture, National Agricultural Statistics Service (USDA, 2015) respectively. A total of 19,365 laboratory-confirmed salmonellosis, caused by *S*. Typhimurium, *S*. Enteritidis, *S*. Newport, and *S*. Javiana were reported in the FoodNet sites from 2004 through 2010. The authors [38] included 14,297 of 19,365 *Salmonella*-infected patients in the analysis based on the presence of valid zip codes (n = 2343 zip codes) available in the Census data and 1,817 (12.7%) *S*. Javiana infections were identified. *S*. Javiana infection was predominantly observed in white (n = 1165, 64.1%), Non-Hispanic (n = 1178, 64.8%) individuals, and among children aged 0 to 4 years (n = 683, 37.6%). This study established several community-level agricultural and environmental factors that were associated with the rate of salmonellosis. Although the rate of salmonellosis varied by serotype in each state, some common trends were identified. The presence of animal feeding operations such as broiler chicken, cattle, dairy, and hog operations were significantly associated with the increasing rate of *Salmonella* infection, as described by Shaw et al. [38]. For instance, in Maryland, *S*. Javiana infection the rate was two times higher [IRR = 2.04 (95% CI 1.29, 3.23)] in the areas where broiler chicken operations were present compared to those without.

#### *S*. Javiana infection in hospital environment

Rathore et al. [22] identified the presence of salmonellosis, including *S*. Javiana infection in a tertiary care center in Florida [22]. The purpose of the study was to identify *Salmonella* serotypes responsible for the most illness. A total of 433 cases of human NTS infection, comprised of 35 different serotypes, were isolated from stool samples of the patient in a tertiary care center from 1986 to 1992. *S*. Javiana (n = 126, 29%) was the most prevalent serotype among all *Salmonella* serotypes. The study conducted by Rathore et al. [22] did not aim to find out the exact source of *S*. Javiana infection, however, they reported the frequency of NTS infection and an abundance of *S*. Javiana serotype at hospital settings. This study [22] was selected for this systematic review as the authors demonstrated that hospital settings could potentially provide a favorable environment for the survival of *Salmonella* and the transmission of NTS infection to human.

Elward et al. [36] conducted a study to identify the source of an outbreak of *S*. Javiana infection at St. Louis Children’s Hospital in St. Louis, Missouri, in 2003. A case-control study of 101 culture-confirmed *S*. Javiana cases and 104 controls was performed. Among the 101 cases, 14% were employees at the food and nutrition department, 44% were hospital employees who worked in departments excluding food and nutrition, 14% were the employees of a neighboring hospital, 16% (n = 16) were the visitors, 9% were hospital affiliated university’s employees, and 4% were patients. Controls were randomly selected from the hospital employees who ate at the cafeteria from May 30 through June 4, 2003, and had no gastroenteritis symptoms after May 1, 2003. Cases, as well as controls, were interviewed using a food questionnaire. A total of 29 food items, most of which were from the salad bar, were more likely to have been consumed by cases compared to controls. To conduct multivariable analysis, Elward et al. [36] defined a composite variable consisting of food items that were consumed from the salad bar. The multivariable analysis revealed that the cases were more likely to have consumed salad bar foods compared to controls [OR_adj_ = 5.3 (95% CI 2.3, 12.1)]. The multivariable model, consisting of date of food consumption in cafeteria as a covariate in addition to food exposure, showed that the consumption of food at the cafeteria on May 28 [OR_adj_ = 9.4 (95% CI 1.8, 49.5)], May 30 [OR_adj_ = 3.6 (95% CI 1.0, 12.7)], and June 3 [OR_adj_ = 4.0 (95% CI 1.4, 11.3)] was significantly associated with the *S*. Javiana infection. Twenty-six of 101 cases consumed only one meal in the hospital cafeteria. Among these 26 cases, 24 cases consumed foods from the salad bar. To identify the source of the *S*. Javiana infection, 123 environmental samples of cafeteria including swabs of cutting boards, food processors, knives, ice-cream machines, and meat thermometer, as well as 84 food samples from the cafeteria were collected on the day the outbreak was reported. All of the environmental and food samples were found to be negative upon laboratory testing. Interviews with the symptomatic food-handlers revealed that many of them helped with replenishing food and ice in the salad bar. Elward et al. [36] concluded that the symptomatic food-handlers might have played a role in the spread the *S*. Javiana infections in the Missouri Children’s hospital.

## Discussion

This systematic review summarizes the risk factors, including food and non-foodborne exposures associated with *S*. Javiana infections reported to date. Consumption of tomatoes [35, 37, 39], cheese [11, 33], paprika-spiced potato chips [21], watermelons [34], and drinking well water [6] were identified as potential vehicles of *S*. Javiana infection. Animal exposures, including exposure to reptile or amphibian, and animal feeding operations, were also identified as potential risk factors for human *S*. Javiana infection [6, 24, 38]. Lastly, the current review also describes the presence of *S*. Javiana infection in healthcare settings [22, 36]. Although *S*. Javiana infection may occur to any age group [6, 11, 22, 24, 33–39], this systematic review highlights the increased incidence in children aged 13 years or younger [6, 21, 34, 36, 38].

Dairy products, particularly cheese are known to transmit many foodborne infections [40–45], [11, 33]. Several potential factors, including cheese preparation using contaminated and/or unpasteurized dairy products, as well as food handling procedures in cheese shredding facility, may contribute to the spread of cheese-borne infections [46, 47]. This systematic review also identified the consumption of fresh produce such as raw tomatoes [35, 37, 39] and fresh watermelon [34] as risk factors for *S*. Javiana infection. A study identified that 2.7% (n = 1,616 out of 60,000 total patients) of the patients, who were diagnosed with NTS infection during nine outbreaks from 1990 to 2004, was reported to have consumed tomatoes [48]. In addition to tomatoes, melons are often associated with foodborne infections, including salmonellosis [49]. Both melons and tomatoes are a field-grown and, thus, the surface of these fruits can be contaminated with dust particles containing feces from birds, and domestic and wild animals [50], [51]. Several studies have been able to isolate *Salmonella* from the surface of watermelons and cantaloupes that were collected directly from the field [49, 52]. The bacteria present on melon or tomato skin can spread into the interior surface during the slicing process [53], [54]. Consumption of these fruits in raw form may increase the likelihood of spreading foodborne illness. Also, melons are often sold as pre-cut, which could provide an additional opportunity for contamination. Both watermelons and tomatoes may be contaminated by *Salmonella* at any stage from growing, to storage at the farms, to the kitchen. Studies suggest that irrigation with contaminated water helps bacteria to colonize and contaminate the fruits and vegetables in the pre-harvest stage [55, 56]. Additionally, *Salmonella* can enter plants and colonize internally through the roots, flowers, stem scar, and even minor cracks in fruit skin [55]. It is still not clear, however, whether the colonization is carried over through generations by infected seeds [57]. Fruits and vegetables can also be contaminated during the post-harvest stage. Many factors, including the improper technique used in the processing facility, inappropriate handling, and inadequate cold storage during transport, at the retail stores, or in kitchens may provide potential opportunity to contaminate fruits and vegetables [49, 58, 59]. Regardless of the source of contamination, microorganisms can multiply rapidly if fruits and vegetables are kept at room temperature for an extended time [60, 61]. Cross-contamination may occur at the processing facility while washing fruits and vegetables in the common wash tank [37, 60, 61], for example, fruits with soft skin, including tomatoes, may absorb contaminated water this way. Despite the use of a highly concentrated chlorine solution, eradication of *Salmonella* from the interior of tomatoes is impossible without cooking [61].

In addition to fruits, spices and herbs were also described as risk factors of *S*. Javiana infection in human [21], [62]. *Salmonella* spp. are known to survive for a prolonged time on the dry surface of foods and spices due to its ability to tolerate desiccation [63, 64]. NTS infection has been associated with numerous dry herbs and spices such as paprika, black pepper, red pepper, and white pepper [21], [62].

In the current systematic review, consumption of well water was also identified as one of the risk factors of *S*. Javiana infection [6]. Several studies have demonstrated the presence of other *Salmonella* serotypes, including *S*. Poona and *S*. Typhimurium in well water [65, 66]. Water is not considered to be the primary source of *Salmonella* because the presence of *Salmonella* in water is often due to fecal contamination [67]. Dust particles, as well as compost contaminated with animal excreta, can contaminate. Additionally, water sources may become contaminated due to improperly treated sewage and stormwater run-off. *Salmonella* can survive for several months in rivers and streams due to its ability to tolerate extreme conditions, including acidity, water turbidity, and temperature changes [68–70]. Like other enteric illness, humans can acquire salmonellosis, during recreational water exposure. In fact, several studies have demonstrated the ability to isolate the same microorganism that causes diarrhea in swimmers from the swimming water itself [71], [72].

Recently, a study conducted by Shaw et al. reported broiler chicken operations as a risk factor of human NTS infection caused by *S*. Javiana serotype [38]. Likewise, several studies demonstrated contact with farm animals could be a major route of transmission of NTS infection by different *Salmonella* serotypes, including *S*. Typhimurium, *S*. Montevideo, and *S*. Newport [73, 74]. The current review also summarized several articles providing evidence that reptile, as well as amphibian exposure, is an important risk factor for *S*. Javiana infections [6, 24]. Similarly, several studies suggest direct or indirect exposure to reptiles (such as turtles, iguanas, lizards, and snakes) and amphibians (such as frogs, and toads) were strongly associated with the NTS infection caused by many *Salmonella* serotypes [75, 76], [77], [78], [79], [80–83]. Reptile, as well as amphibian associated NTS infection, is an ongoing public health concern [24, 79, 84, 85]. There are approximately 74,000 (6% of the total 1.2 million cases) *Salmonella* infections each year in the United States due to the reptile and amphibian exposure [85].

Keeping reptiles as pets has been increasing in the United States since 1991, and the same trend has resulted in a concurrent increase in reptile-associated human NTS infections [79, 86]. Reptiles are known to carry several *Salmonella* serotypes and may shed the pathogen either continuously or intermittently in their feces [79]. Turtles, an asymptomatic carrier of *Salmonella*, is considered as a key source of NTS infection in the USA since the 1970s [87]. Handling, kissing, as well as direct contact with turtle’s feces, could be potential routes of NTS infection in human [82, 83]. The Food and Drug Administration (FDA) banned the sale of small turtles (shell length less than 4 inches or 10.16 cm) in 1975, and since then there has been an estimated decrease of 100,000 human salmonellosis cases each year [87]. Several studies have suggested the transmission of NTS infection through indirect contact with animals, for instance, visiting a turtle keeper or receiving a blood transfusion from a donor with a pet snake [80, 83].

In addition to reptiles, amphibians are also known carriers of several *Salmonella* serotypes [88]. Amphibians, including snails, frogs, and toads, have been associated with the transmission of *Salmonella* infections in human [24, 85, 89]. Recently, a study described that aquatic frogs, namely, African dwarf frogs, have been associated with *Salmonella* outbreak predominantly occurring in children during 2008 through 2011 in the United States [90]. In the same outbreak, an estimated 29% of the patients were hospitalized for one to nine days, with a median of four days. *Salmonella* is known to survive for an extended period (approximately up to 30 months) on inanimate surfaces due to its tolerance response against the extremely acidic environment, desiccation, and low oxygen tension [63, 64, 85]. Thus, minimal exposure to surfaces contaminated by reptile’s or amphibian feces could potentially lead to salmonellosis in human [84, 86, 91]. NTS infection has also been found to be transmitted through indirect exposure to reptiles and amphibians, such as through contact with aquariums at pet stores, schools, or child care centers, or friend’s or relative’s home [92]. Therefore, aquariums should not be cleaned in the kitchen sinks, bathroom sinks, and bathtubs, since that could lead to cross-contamination [88]. Additionally, children less than five years should not be allowed to come in contact with amphibians, particularly, frogs, in child care facilities or schools [93]. Likewise, all high-risk persons, including children below five years of age, the elderly, pregnant women, and immunocompromised persons, should not be exposed to African dwarf frogs and associated water [94]. It has been observed that public awareness regarding reptile and amphibian-associated salmonellosis is low [90]. A recent study revealed that only 38% of patients interviewed were aware of the risk of *Salmonella* illness from exposure to reptiles, and only 18% were familiar with the risk of *Salmonella* infection from amphibians [90]. Although, CDC’s Healthy Pets Healthy People Web site (https://www.cdc.gov/healthypets/index.html) [95] published educational flyers on *Salmonella* infections from animal contact, public awareness of the risk illness from reptiles and amphibians has not expanded as expected. Additional public awareness activities are needed so the public takes precautions while handling amphibians and reptiles.

The current systematic review also provides evidence on *S*. Javiana infection in hospital settings, including tertiary care centers in Florida [22] and Missouri [36]. Person to person contact and foodborne transmission are known as the most common sources of salmonellosis in hospital settings [96]. Several studies reported the spreading of foodborne hospital-acquired infections due to inadequate personal hygiene of food handlers, negligence in proper hygiene practices while preparing and distributing foods among patients [97, 98]. In general, outbreaks in hospital settings need special attention since patients may acquire coincident infections which may pose a great threat to their life. All necessary measures should be taken into consideration to avoid hospital outbreaks of Salmonellosis.

The current study systematically presents the sources of *S*. Javiana infection in humans based on the available evidence in the literature. *S*. Javiana is reported to be transmitted by both animal exposure and/or consumption of contaminated food. It has been observed that *S*. Javiana infection was mostly observed during July through October [17]. The seasonality may indicate the relationship with the abundance of its reservoirs, for example, reptiles and amphibians, in the environment. Similarly, fruits (such as watermelons) and vegetables (tomatoes) can be contaminated with *S*. Javiana by the contaminated irrigation water or dust particle-containing animal’s excreta. In our current study, the majority of the reports were the cases of food-associated *S*. Javiana infection (7 out of 12). Three articles identified animal exposure, while two studies reported infection in the hospital setting by *S*. Javiana. As it is evident from this data that foodborne exposures continues to be reported as the major source of *S*. Javiana infection, however, a report from FSIS clearly shows that only 30 *S*. Javiana positive food sample were identified from 500,000 HACCP samples analyzed between 1998 and 2009 [7], and the agency *“…found no significant correlations with the FSIS-regulated products analyzed and human illnesses from* S. *Javiana*” [7]. Consistent with FSIS report, our study also found only one article (Shaw et al. [38]) that associated potential FSIS regulated product with *S*. Javiana. Given the body of evidence, it is hard to compare the risk potentials of various sources of *S*. Javiana infection. However, it is plausible to assume that fruits (such as, watermelons) and vegetables (tomatoes), irrigation water used for fresh produce agriculture along with exposures to reptiles and amphibians endemic in Southeast U.S. are the major sources of this serotype.

## Limitations

Although this systematic review used the best available evidence, there are some limitations. The exposure definition, outcome measurement, and potential confounding factors were defined differently across the individual studies. The current systematic review attempted to capture all relevant information available to date, however, there is the possibility of bias. Studies that were published in languages other than English were excluded from the systematic review. Moreover, the studies that resulted in a non-significant association between risk factors and *S*. Javiana are less likely to be published and, therefore, this systematic review is not free from publication bias.

Furthermore, most of the individual studies [6, 11, 24, 34–37, 39], that were included in the current systematic review are subject to recall bias, since cases are more likely to have accurately reported the exposure information compared to the controls groups. In general, cases are more likely to remember information correctly due to their health concerns compared to control or non-disease population. In addition to recall bias, most of the included individual studies [6, 11, 24, 34–37, 39] may contain interview bias. There are a few studies discussed in this systematic review where may have selection bias may be present. For example, the study by Srikantiah et al. reports a participation rate of only 34% in the first interview [39]. This study received a quality appraisal score of 6 (fair) as the study used a web-based investigation tool limiting responses to only a few attendees of Transplant Games with a registered email address in the first interview [39]. In another study [37], the authors did not discuss if any exclusion criteria were used to select the control groups [37]. This discrepancy may result in selection bias in their study. Clarkson et al. [6] selected the controls from the respondents of the FoodNet Population Survey, conducted from Georgia and Tennessee in 2002 and 2003. Selection of controls from previous years may introduce selection bias in their study as they may not be representative of the population that gave rise to the cases. Accordingly, both of the studies (Clarkson et al. and Hedberg et al.) were rated as “fair” with a cumulative score of 6. In general, systematic reviews may introduce database bias if the authors relied on limited databases. However, this systematic review minimizes the database bias since three relevant databases, namely, PubMed, Web of Science, and MMWR, were used for the literature search.

## Conclusions

In conclusion, this systematic review provides comprehensive evidence of potential risk factors with *S*. Javiana infection in humans reported to date. Available evidence suggests that foodborne, as well as non-foodborne exposure, contribute substantially to the transmission of *Salmonella* serotype Javiana. Fresh produce (tomatoes and watermelons), herbs (paprika-spice), and dairy products (cheese) were reported to be associated with *S*. Javiana infection in humans. In addition to the foodborne exposure, this review suggests that human *S*. Javiana infection is associated with consumption of contaminated well water. Animal contact has also been found to be significantly associated with *S*. Javiana infection in human. The current review identifies the risk factors associated with *S*. Javiana infection. This may be helpful in the risk management of *S*. Javiana infection and transmission. Most of these risk factors mainly arise from improper management practices and are correctable, therefore, strategies to prevent the occurrence of human NTS infection, including *S*. Javiana infection, should include a focus on the consumption of treated drinking water, safe animal contact, as well as safe food processing and handling procedures. Although there has been substantial research on the risk factors and the mode of transmission of *S*. Javiana infection, the rate of illness is still growing. The reason for the increase of *S*. Javiana associated infection is not known. Therefore, further investigation is still needed to gain insight into this matter to mitigate *S*. Javiana infection and associated healthcare costs.

## Supporting information

S1 Fig PRISMA checklist. (DOC)

## References

[1] Majowicz SE, Musto J, Scallan E, Angulo FJ, Kirk M, O'Brien SJ, et al The global burden of nontyphoidal *Salmonella* gastroenteritis. Clinical infectious diseases: an official publication of the Infectious Diseases Society of America. 2010;50(6):882–9. Epub 2010/02/18. 10.1086/650733 . 20158401

[2] Scallan E, Hoekstra RM, Angulo FJ, Tauxe RV, Widdowson MA, Roy SL, et al Foodborne illness acquired in the United States—major pathogens. Emerging infectious diseases. 2011;17(1):7–15. Epub 2011/01/05. 10.3201/eid1701.P11101 PubMed PMID: 21192848; PubMed Central PMCID: PMC3375761. 21192848 PMC3375761

[3] Tindall B, Grimont P, Garrity G, Euzeby J. Nomenclature and taxonomy of the genus *Salmonella*. International journal of systematic and evolutionary microbiology. 2005;55(1):521–4. 15653930 10.1099/ijs.0.63580-0

[4] Braun SD, Ziegler A, Methner U, Slickers P, Keiling S, Monecke S, et al Fast DNA serotyping and antimicrobial resistance gene determination of *Salmonella* enterica with an oligonucleotide microarray-based assay. PloS one. 2012;7(10):e46489 Epub 2012/10/12. 10.1371/journal.pone.0046489 23056321 PMC3464306

[5] CDC. National Salmonella Surveillance: Annual Data Summary, 2011. Atlanta, Georgia: US Department of Health and Human Services, Centers for Disease Control and Prevention, 2013. 2013.

[6] Clarkson LS, Tobin-D'Angelo M, Shuler C, Hanna S, Benson J, Voetsch AC. Sporadic *Salmonella* enterica serotype Javiana infections in Georgia and Tennessee: a hypothesis-generating study. Epidemiology and infection. 2010;138(3):340–6. Epub 2009/09/03. 10.1017/S0950268809990586 . 19723360

[7] FSIS. A comparison of Salmonella serotype incidence in FSIS-regulated products and salmonellosis cases. USDA-FSIS Report (web resource). 2012.

[8] Miller RA, Wiedmann M. The Cytolethal Distending Toxin Produced by Nontyphoidal *Salmonella* Serotypes Javiana, Montevideo, Oranienburg, and Mississippi Induces DNA Damage in a Manner Similar to That of Serotype Typhi. MBio. 2016;7(6). Epub 2016/12/22. 10.1128/mBio.02109-16 27999166 PMC5181781

[9] Miller RA, Betteken MI, Guo X, Altier C, Duhamel GE, Wiedmann M. The Typhoid Toxin Produced by the Nontyphoidal Salmonella enterica Serotype Javiana Is Required for Induction of a DNA Damage Response In Vitro and Systemic Spread In Vivo. MBio. 2018;9(2). Epub 2018/03/29. 10.1128/mBio.00467-18 29588404 PMC5874915

[10] Cheng RA, Eade CR, Wiedmann M. Embracing Diversity: Differences in Virulence Mechanisms, Disease Severity, and Host Adaptations Contribute to the Success of Nontyphoidal Salmonella as a Foodborne Pathogen. Front Microbiol. 2019;10:1368 Epub 2019/07/19. 10.3389/fmicb.2019.01368 31316476 PMC6611429

[11] Hedberg CW, Korlath JA, D'Aoust JY, White KE, Schell WL, Miller MR, et al A multistate outbreak of *Salmonella* Javiana and *Salmonella* Oranienburg infections due to consumption of contaminated cheese. JAMA. 1992;268(22):3203–7. Epub 1992/12/09. . 1433759

[12] Toth B, Bodager D, Hammond R, Stenzel S, Adams J, Kass-Hout T, et al Outbreak of Salmonella serotype Javiana infections-Orlando, Florida, June 2002. Morbidity and Mortality Weekly Report. 2002;51(31):683–4. 12233909

[13] Gracia Jover S, Perez Canas C, Vavken E. [A case of meningitis cause by *Salmonella* Javiana]. Revista latinoamericana de microbiologia y parasitologia. 1967;9(1):15–7. Epub 1967/01/01. . 5245491

[14] Grossmann E, Hancke S. Polycystic liver disease, complicated by *Salmonella* infection. Scandinavian journal of gastroenterology. 1996;31(9):940–2. Epub 1996/09/01. 10.3109/00365529609052006 . 8888445

[15] Lee J, McLeod M, Meyers W, Arthur J, Corey G. Successful laparoscopic management of perforated gallbladder associated with *Salmonella* Javiana infection. North Carolina medical journal. 1992;53(11):594–5. 1436154

[16] Gordon MA. *Salmonella* infections in immunocompromised adults. The Journal of infection. 2008;56(6):413–22. Epub 2008/05/14. 10.1016/j.jinf.2008.03.012 . 18474400

[17] CDC. An Atlas of Salmonella in the United States, 1968–2011. Laboratory-based Enteric Disease Surveillance, Atlanta, Georgia. 2015.

[18] Gordon MA, Banda HT, Gondwe M, Gordon SB, Boeree MJ, Walsh AL, et al Non-typhoidal salmonella bacteraemia among HIV-infected Malawian adults: high mortality and frequent recrudescence. AIDS. 2002;16(12):1633–41. 10.1097/00002030-200208160-00009 12172085

[19] Walsh AL, Phiri AJ, Graham SM, Molyneux EM, Molyneux ME. Bacteremia in febrile Malawian children: clinical and microbiologic features. The Pediatric infectious disease journal. 2000;19(4):312–9. 10.1097/00006454-200004000-00010 10783021

[20] Atkinson N, Carter MC, Wollaston JM, Wall M. THE OCCURRENCE OF *SALMONELLA* TYPES IN AUSTRALIA. Australian Journal of Experimental Biology & Medical Science. 1953;31(5). 10.1038/icb.1953.50 13115307

[21] Lehmacher A, Bockemuhl J, Aleksic S. Nationwide outbreak of human salmonellosis in Germany due to contaminated paprika and paprika-powdered potato chips. Epidemiology and infection. 1995;115(3):501–11. Epub 1995/12/01. 10.1017/s0950268800058660 8557082 PMC2271603

[22] Rathore MH, Jenkins SG, Williams E. Epidemiology of nontyphoidal salmonellae at a tertiary care center in northeast Florida. Southern medical journal. 1995;88(8):840–2. Epub 1995/08/01. . 7631210 10.1097/00007611-199508000-00009

[23] CDC. Preliminary FoodNet data on the incidence of infection with pathogens transmitted commonly through food—10 sites, United States, 2004. MMWR Morbidity and mortality weekly report. 2005;54(14):352 15829864

[24] Srikantiah P, Lay JC, Hand S, Crump JA, Campbell J, Van Duyne MS, et al *Salmonella* enterica serotype Javiana infections associated with amphibian contact, Mississippi, 2001. Epidemiology and infection. 2004;132(2):273–81. Epub 2004/04/06. 10.1017/s0950268803001638 15061502 PMC2870103

[25] de Souza LS, Godwin JC, Renshaw MA, Larson E. Environmental DNA (eDNA) Detection Probability Is Influenced by Seasonal Activity of Organisms. PloS one. 2016;11(10):e0165273 Epub 2016/10/25. 10.1371/journal.pone.0165273 27776150 PMC5077074

[26] Moher D, Liberati A, Tetzlaff J, Altman DG, The PG. Preferred Reporting Items for Systematic Reviews and Meta-Analyses: The PRISMA Statement. PLoS Med. 2009;6(7):e1000097 10.1371/journal.pmed.1000097 19621072 PMC2707599

[27] Mukherjee N. Source attribution, antibiotic resistance and virulence properties of *Salmonella* serotypes isolated from clinically disgnosed human salmonellosis cases from Tennessee. Doctoral dissertation, University of Memphis. 2018.

[28] Liu F, Li J, Wu F, Zheng H, Peng Q, Zhou H. Altered composition and function of intestinal microbiota in autism spectrum disorders: a systematic review. Translational psychiatry. 2019;9(1):43 10.1038/s41398-019-0389-6 30696816 PMC6351640

[29] Wells GA, Shea B, O'Connell D, Peterson J, Welch V, Losos M, et al The Newcastle-Ottawa Scale (NOS) for assessing the quality of nonrandomised studies in meta-analyses. NOS Manual. 2019; [www.ohri.ca/programs/clinical_epidemiology/oxford.asp] (Accessed July 15, 2019).

[30] Balle CM, Jeppesen AN, Christensen S, Hvas AM. Platelet Function During Extracorporeal Membrane Oxygenation in Adult Patients: A Systematic Review. Front Cardiovasc Med. 2018;5:157 Epub 2018/11/27. 10.3389/fcvm.2018.00157 30474031 PMC6237979

[31] NHLBI. National Heart Lung Blood Institute: Quality Assessment Tool for Case Series Studies. 2019; Available online at: https://www.nhlbi.nih.gov/health-topics/study-quality-assessment-tools (Accessed July 15, 2019).

[32] NHLBI. Background: Development and Use of Study Quality Assessment Tools. 2019; Available online at: www.nhlbi.nih.gov/node/80102 (Accessed July 15, 2019).

[33] Alley RD, Pijoan M. *Salmonella* Javiana Food Infection. The Yale journal of biology and medicine. 1942;15(2):229–39. Epub 1942/12/01. 21434061 PMC2601257

[34] Blostein J. An outbreak of *Salmonella* javiana associated with consumption of watermelon. J Environ Health. 1993;56(1):29–31.

[35] Corby R, Lanni V.., Kistler V.. Outbreaks of *Salmonella* infections associated with eating Roma tomatoes United States and Canada, 2004. MMWR Morbidity and mortality weekly report. 2005;54(13):325–8. 15815562

[36] Elward A, Grim A, Schroeder P, Kieffer P, Sellenriek P, Ferrett R, et al Outbreak of *Salmonella* javiana infection at a children's hospital. Infection control and hospital epidemiology. 2006;27(6):586–92. Epub 2006/06/07. 10.1086/504935 . 16755478

[37] Hedberg CW, Angulo FJ, White KE, Langkop CW, Schell WL, Stobierski MG, et al Outbreaks of salmonellosis associated with eating uncooked tomatoes: implications for public health. The Investigation Team. Epidemiology and infection. 1999;122(3):385–93. Epub 1999/08/25. 10.1017/s0950268899002393 10459640 PMC2809631

[38] Shaw KS, Cruz-Cano R, Jiang C, Malayil L, Blythe D, Ryan P, et al Presence of animal feeding operations and community socioeconomic factors impact salmonellosis incidence rates: An ecological analysis using data from the Foodborne Diseases Active Surveillance Network (FoodNet), 2004–2010. Environmental research. 2016;150:166–72. Epub 2016/06/13. 10.1016/j.envres.2016.05.049 . 27290657

[39] Srikantiah P, Bodager D, Toth B, Kass-Hout T, Hammond R, Stenzel S, et al Web-based investigation of multistate salmonellosis outbreak. Emerging infectious diseases. 2005;11(4):610–2. Epub 2005/04/15. 10.3201/eid1104.040997 15829202 PMC3320341

[40] CDC. *Salmonella* typhimurium infection associated with raw milk and cheese consumption—Pennsylvania, 2007. MMWR Morbidity and mortality weekly report. 2007;56(44):1161 17989645

[41] Cody SH, Abbott SL, Marfin AA, Schulz B, Wagner P, Robbins K, et al Two outbreaks of multidrug-resistant *Salmonella* serotype typhimurium DT104 infections linked to raw-milk cheese in Northern California. Jama. 1999;281(19):1805–10. 10.1001/jama.281.19.1805 10340367

[42] Costard S, Espejo L, Groenendaal H, Zagmutt FJ. Outbreak-Related Disease Burden Associated with Consumption of Unpasteurized Cow’s Milk and Cheese, United States, 2009–2014. Emerging infectious diseases. 2017;23(6):957 10.3201/eid2306.151603 28518026 PMC5443421

[43] Dominguez M, Jourdan-Da Silva N, Vaillant V, Pihier N, Kermin C, Weill F-X, et al Outbreak of *Salmonella* enterica serotype Montevideo infections in France linked to consumption of cheese made from raw milk. Foodborne pathogens and disease. 2009;6(1):121–8. 10.1089/fpd.2008.0086 19072083

[44] Ellis A, Preston M, Borczyk A, Miller B, Stone P, Hatton B, et al A community outbreak of *Salmonella* berta associated with a soft cheese product. Epidemiology & Infection. 1998;120(1):29–35. 9528815 10.1017/s0950268897008376 PMC2809346

[45] Magalhaes R, Almeida G, Ferreira V, Santos I, Silva J, Mendes MM, et al Cheese-related listeriosis outbreak, Portugal, March 2009 to February 2012. Euro surveillance: bulletin Europeen sur les maladies transmissibles = European communicable disease bulletin. 2015;20(17). Epub 2015/05/09. 10.2807/1560-7917.es2015.20.17.21104 . 25955775

[46] Choi K-H, Lee H, Lee S, Kim S, Yoon Y. Cheese Microbial Risk Assessments—A Review. Asian-Australasian journal of animal sciences. 2016;29(3):307 10.5713/ajas.15.0332 26950859 PMC4811779

[47] Kousta M, Mataragas M, Skandamis P, Drosinos EH. Prevalence and sources of cheese contamination with pathogens at farm and processing levels. Food control. 2010;21(6):805–15.

[48] Voetsch AC, Van Gilder TJ, Angulo FJ, Farley MM, Shallow S, Marcus R, et al FoodNet estimate of the burden of illness caused by nontyphoidal *Salmonella* infections in the United States. Clinical Infectious Diseases. 2004;38(Supplement 3):S127–S34. 15095181 10.1086/381578

[49] Walsh KA, Bennett SD, Mahovic M, Gould LH. Outbreaks associated with cantaloupe, watermelon, and honeydew in the United States, 1973–2011. Foodborne Pathog Dis. 2014;11(12):945–52. Epub 2014/11/20. 10.1089/fpd.2014.1812 25407556 PMC4627691

[50] Kumar GD, Williams RC, Al Qublan HM, Sriranganathan N, Boyer RR, Eifert JD. Airborne soil particulates as vehicles for *Salmonella* contamination of tomatoes. International journal of food microbiology. 2017;243:90–5. Epub 2016/12/31. 10.1016/j.ijfoodmicro.2016.12.006 . 28038335

[51] Gruszynski K, Pao S, Kim C, Toney D, Wright K, Ross PG, et al Evaluating wildlife as a potential source of *Salmonella* serotype Newport (JJPX01.0061) contamination for tomatoes on the eastern shore of Virginia. Zoonoses and public health. 2014;61(3):202–7. Epub 2013/06/19. 10.1111/zph.12061 . 23773825

[52] CDC. Multistate outbreak of *Salmonella* poona infections—United States and Canada, 1991. MMWR Morbidity and mortality weekly report. 1991;40(32):549 1861671

[53] Danyluk MD, Friedrich LM, Schaffner DW. Modeling the growth of *Listeria monocytogenes* on cut cantaloupe, honeydew and watermelon. Food microbiology. 2014;38:52–5. Epub 2013/12/03. 10.1016/j.fm.2013.08.001 . 24290625

[54] Lin C-m Wei C-i. Transfer of *Salmonella* montevideo onto the interior surfaces of tomatoes by cutting. Journal of Food Protection. 1997;60(7):858–62. 10.4315/0362-028X-60.7.858 31026878

[55] Guo X, van Iersel MW, Chen J, Brackett RE, Beuchat LR. Evidence of association of salmonellae with tomato plants grown hydroponically in inoculated nutrient solution. Applied and environmental microbiology. 2002;68(7):3639–43. Epub 2002/06/29. 10.1128/AEM.68.7.3639-3643.2002 12089054 PMC126780

[56] Hintz LD, Boyer RR, Ponder MA, Williams RC, Rideout SL. Recovery of *Salmonella enterica* Newport introduced through irrigation water from tomato (Lycopersicum esculentum) fruit, roots, stems, and leaves. HortScience. 2010;45(4):675–8.

[57] Guo X, Chen J, Brackett RE, Beuchat LR. Survival of *Salmonellae* on and in tomato plants from the time of inoculation at flowering and early stages of fruit development through fruit ripening. Applied and environmental microbiology. 2001;67(10):4760–4. 10.1128/AEM.67.10.4760-4764.2001 11571182 PMC93229

[58] Castillo A, Mercado I, Lucia LM, Martinez-Ruiz Y, Ponce de Leon J, Murano EA, et al *Salmonella* contamination during production of cantaloupe: a binational study. J Food Prot. 2004;67(4):713–20. Epub 2004/04/16. 10.4315/0362-028x-67.4.713 . 15083723

[59] Arah IK, Ahorbo GK, Anku EK, Kumah EK, Amaglo H. Postharvest Handling Practices and Treatment Methods for Tomato Handlers in Developing Countries: A Mini Review. Advances in Agriculture. 2016;2016.

[60] Wei C, Huang T, Kim J, Lin W, TAMPLlN M, Bartz J. Growth and survival of *Salmonella* montevideo on tomatoes and disinfection with chlorinated water. Journal of Food Protection. 1995;58(8):829–36. 10.4315/0362-028X-58.8.829 31137395

[61] Zhuang R, Beuchat L, Angulo F. Fate of *Salmonella* Montevideo on and in raw tomatoes as affected by temperature and treatment with chlorine. Applied and environmental microbiology. 1995;61(6):2127–31. 7793934 10.1128/aem.61.6.2127-2131.1995 PMC167485

[62] CDC. Investigation update: Multistate outbreak of human *Salmonella* Montevideo infections. Centers for Disease Control and Prevention Atlanta, GA; 2010.

[63] Hiramatsu R, Matsumoto M, Sakae K, Miyazaki Y. Ability of Shiga toxin-producing *Escherichia coli* and *Salmonella* spp. to survive in a desiccation model system and in dry foods. Applied and environmental microbiology. 2005;71(11):6657–63. 10.1128/AEM.71.11.6657-6663.2005 16269694 PMC1287607

[64] Ristori CA, dos Santos Pereira MA, Gelli DS. Behavior of Salmonella Rubislaw on ground black pepper (Piper nigrum L.). Food Control. 2007;18(3):268–72.

[65] Traoré O, Nyholm O, Siitonen A, Bonkoungou IJO, Traoré AS, Barro N, et al Prevalence and diversity of *Salmonella enterica* in water, fish and lettuce in Ouagadougou, Burkina Faso. BMC microbiology. 2015;15(1):151. 26228572 10.1186/s12866-015-0484-7 PMC4521495

[66] McFeters GA, Bissonnette GK, Jezeski JJ, Thomson CA, Stuart DG. Comparative survival of indicator bacteria and enteric pathogens in well water. Applied microbiology. 1974;27(5):823–9. Epub 1974/05/01. 4598219 10.1128/am.27.5.823-829.1974 PMC380150

[67] Abulreesh HH. Salmonellae in the environment. Salmonella-Distribution, adaptation, control measures and molecular technologies: InTech; 2012.

[68] De Rezende CE, Mallinson E, Gupte A, Joseph S. *Salmonella* spp. are affected by different levels of water activity in closed microcosms. Journal of Industrial Microbiology and Biotechnology. 2001;26(4):222–5. 11464270 10.1038/sj.jim.7000116

[69] Leyer G, Johnson E. Acid adaptation promotes survival of *Salmonella* spp. in cheese. Applied and environmental microbiology. 1992;58(6):2075–80. 1622286 10.1128/aem.58.6.2075-2080.1992 PMC195729

[70] Moore BC, Martinez E, Gay JM, Rice DH. Survival of *Salmonella* enterica in freshwater and sediments and transmission by the aquatic midge Chironomus tentans (Chironomidae: Diptera). Applied and environmental microbiology. 2003;69(8):4556–60. 10.1128/AEM.69.8.4556-4560.2003 12902242 PMC169145

[71] Sanborn M, Takaro T. Recreational water–related illness. Canadian Family Physician. 2013;59(5):491–5. 23673583 PMC3653650

[72] Wiedenmann A, Krüger P, Dietz K, López-Pila JM, Szewzyk R, Botzenhart K. A randomized controlled trial assessing infectious disease risks from bathing in fresh recreational waters in relation to the concentration of *Escherichia coli*, intestinal enterococci, Clostridium perfringens, and somatic coliphages. Environmental health perspectives. 2006;114(2):228 10.1289/ehp.8115 16451859 PMC1367836

[73] Cummings KJ, Warnick LD, Davis MA, Eckmann K, Gröhn YT, Hoelzer K, et al Farm animal contact as risk factor for transmission of bovine-associated Salmonella subtypes. Emerging infectious diseases. 2012;18(12):1929 10.3201/eid1812.110831 23171627 PMC3557873

[74] Stevens MP, Humphrey TJ, Maskell DJ. Molecular insights into farm animal and zoonotic *Salmonella* infections. Philosophical Transactions of the Royal Society B: Biological Sciences. 2009;364(1530):2709–23. 10.1098/rstb.2009.0094 PubMed PMID: PMC2865095. 19687040 PMC2865095

[75] Ives AK, Antaki E, Stewart K, Francis S, Jay-Russell MT, Sithole F, et al Detection of *Salmonella enterica* Serovar Montevideo and Newport in Free-ranging Sea Turtles and Beach Sand in the Caribbean and Persistence in Sand and Seawater Microcosms. Zoonoses and public health. 2016 Epub 2016/12/23. 10.1111/zph.12324 . 28009107

[76] CDC. Outbreak of multidrug-resistant *Salmonella* newport—United States, January-April 2002. MMWR Morb Mortal Wkly Rep. 2002a;51(25):545–8. Epub 2002/07/18. . 12118534

[77] Ribas A, Poonlaphdecha S. Wild-Caught and Farm-Reared Amphibians are Important Reservoirs of *Salmonella*, A Study in North-East Thailand. Zoonoses and public health. 2016 Epub 2016/07/01. 10.1111/zph.12286 . 27359101

[78] Woodward DL, Khakhria R, Johnson WM. Human salmonellosis associated with exotic pets. Journal of Clinical Microbiology. 1997;35(11):2786–90. 9350734 10.1128/jcm.35.11.2786-2790.1997 PMC230062

[79] CDC. Reptile-associated salmonellosis—selected states, 1996–1998. MMWR Morb Mortal Wkly Rep. 1999;48(44):1009–13. Epub 1999/11/30. . 10577489

[80] Jafari M, Forsberg J, Gilcher RO, Smith JW, Crutcher JM, McDermott M, et al *Salmonella* Sepsis Caused by a Platelet Transfusion from a Donor with a Pet Snake. New England Journal of Medicine. 2002;347(14):1075–8. 10.1056/NEJMoa021050 . 12362008

[81] Warwick C, Lambiris AJ, Westwood D, Steedman C. Reptile-related salmonellosis. Journal of the Royal Society of Medicine. 2001;94(3):124–6. PubMed PMID: PMC1297927. 10.1177/014107680109400306 11285792 PMC1297927

[82] Harris JR, Neil KP, Behravesh CB, Sotir MJ, Angulo FJ. Recent multistate outbreaks of human *Salmonella* infections acquired from turtles: a continuing public health challenge. Clinical infectious diseases: an official publication of the Infectious Diseases Society of America. 2010;50(4):554–9. Epub 2010/01/21. 10.1086/649932 . 20085463

[83] Stam F, Römkens TEH, Hekker TAM, Smulders YM. Turtle-Associated Human Salmonellosis. Clinical Infectious Diseases. 2003;37(11):e167–e9. 10.1086/379612 14614690

[84] CDC. Reptile-associated salmonellosis—selected states, 1994–1995. MMWR Morb Mortal Wkly Rep. 1995;44(17):347–50. Epub 1995/05/05. . 7715594

[85] Mermin J, Hutwagner L, Vugia D, Shallow S, Daily P, Bender J, et al Reptiles, amphibians, and human *Salmonella* infection: a population-based, case-control study. Clinical Infectious Diseases. 2004;38(Supplement_3):S253–S61. 15095197 10.1086/381594

[86] Mermin J, Hoar B, Angulo FJ. Iguanas and *Salmonella* marina infection in children: a reflection of the increasing incidence of reptile-associated salmonellosis in the United States. Pediatrics. 1997;99(3):399–402. 10.1542/peds.99.3.399 9041295

[87] Cohen ML, Potter M, Pollard R, Feldman RA. Turtle-associated salmonellosis in the United States: effect of public health action, 1970 to 1976. Jama. 1980;243(12):1247–9. 7359680

[88] Bartlett K, Lior H. Small pet aquarium frogs as a source of *Salmonella*. Applied and environmental microbiology. 1977;33(5):1026–9. 879765 10.1128/aem.33.5.1026-1029.1977 PMC170822

[89] Bartlett K. Isolation of *Salmonellae* and other potential pathogens from the freshwater aquarium snail Ampullaria. Applied and environmental microbiology. 1976;31(5):635–9. 818954 10.1128/aem.31.5.635-639.1976 PMC291168

[90] Zarecki SLM, Bennett SD, Hall J, Yaeger J, Lujan K, Adams-Cameron M, et al US outbreak of human *Salmonella* infections associated with aquatic frogs, 2008–2011. Pediatrics. 2013;131(4):724–31. 10.1542/peds.2012-2031 23478862

[91] Friedman CR, Torigian C, Shillam PJ, Hoffman RE, Heltze D, Beebe JL, et al An outbreak of salmonellosis among children attending a reptile exhibit at a zoo. The Journal of pediatrics. 1998;132(5):802–7. 10.1016/s0022-3476(98)70307-5 9602189

[92] Trust T, Bartlett KH, Lior H. Importation of *Salmonellae* with aquarium species. Canadian journal of microbiology. 1981;27(5):500–4. 10.1139/m81-074 7248855

[93] CDC. Compendium of measures to prevent disease associated with animals in public settings, 2011: National Association of State Public Health Veterinarians, Inc. (NASPHV). MMWR Recommendations and reports: Morbidity and mortality weekly report Recommendations and reports/Centers for Disease Control. 2011b;60(RR-04):1. 21546893

[94] CDC. Investigation update: ongoing outbreak of human *Salmonella* Typhimurium infections associated with African dwarf frogs. 2011a.

[95] CDC. Investigation update: ongoing outbreak of human *Salmonella* Typhimurium infections associated with African dwarf frogs. Available at: wwwcdcgov/salmonella/water-frogs-0411/indexhtml. 2012.

[96] Palmer S, Rowe B. Investigation of outbreaks of *Salmonella* in hospitals. Br Med J (Clin Res Ed). 1983;287(6396):891–3. 10.1136/bmj.287.6396.891 PMC1549232 6412874

[97] Lund BM, O'Brien SJ. Microbiological safety of food in hospitals and other healthcare settings. Journal of Hospital Infection. 2009;73(2):109–20. 10.1016/j.jhin.2009.05.017 19732991

[98] Vonberg R-P, Weitzel-Kage D, Behnke M, Gastmeier P. Worldwide Outbreak Database: the largest collection of nosocomial outbreaks. Infection. 2011;39(1):29–34. 10.1007/s15010-010-0064-6 21153042 PMC7100329

